# LZTFL1 inhibits kidney tumor cell growth by destabilizing AKT through ZNRF1-mediated ubiquitin proteosome pathway

**DOI:** 10.1038/s41388-023-02666-x

**Published:** 2023-03-25

**Authors:** Jun Lu, Liang-min Fu, Yun Cao, Yong Fang, Jia-zheng Cao, Yi-hui Pan, Jun-jie Cen, Yan-ping Liang, Zhen-hua Chen, Jin-huan Wei, Yong Huang, Mukhtar Adan Mumin, Quan-hui Xu, Ying-han Wang, Jiang-quan Zhu, Hui Liang, Zhu Wang, Qiong Deng, Wei Chen, Xiao-han Jin, Zhi-ping Liu, Jun-hang Luo

**Affiliations:** 1 Department of Urology, The First Affiliated Hospital of Sun Yat-sen University, Guangzhou, People’s Republic of China; 2 Departments of Internal Medicine and Molecular Biology, UT Southwestern Medical Center, Dallas, TX USA; 3 Department of Pathology, Sun Yat-sen University Cancer Center of Sun Yat-sen University, Guangzhou, People’s Republic of China; 4 Department of Urology, Jiangmen Central Hospital, Jiangmen, Guangdong Province People’s Republic of China; 5 Department of Urology, The First People’s Hospital of Changzhou, Changzhou, Jiangsu People’s Republic of China; 6 Department of Emergency, The First Affiliated Hospital of Sun Yat-sen University, Guangzhou, People’s Republic of China; 7 Department of Urology, Affiliated Longhua People’s Hospital, Southern Medical University, Shenzhen, Guangdong Province People’s Republic of China

**Keywords:** Renal cancer, Cell growth

## Abstract

LZTFL1 is a tumor suppressor located in chromosomal region 3p21.3 that is deleted frequently and early in various cancer types including the kidney cancer. However, its role in kidney tumorigenesis remains unknown. Here we hypothesized a tumor suppressive function of LZTFL1 in clear cell renal cell carcinoma (ccRCC) and its mechanism of action based on extensive bioinformatics analysis of patients’ tumor data and validated it using both gain- and loss-functional studies in kidney tumor cell lines and patient-derive xenograft (PDX) model systems. Our studies indicated that LZTFL1 inhibits kidney tumor cell proliferation by destabilizing AKT through ZNRF1-mediated ubiquitin proteosome pathway and inducing cell cycle arrest at G1. Clinically, we found that LZTFL1 is frequently deleted in ccRCC. Downregulation of LZTFL1 is associated with a poor ccRCC outcome and may be used as prognostic maker. Furthermore, we show that overexpression of LZTFL1 in PDX via lentiviral delivery suppressed PDX growth, suggesting that re-expression of LZTFL1 may be a therapeutic strategy against ccRCC.

## Introduction

Renal cell carcinoma (RCC) is the sixth most frequently diagnosed cancer in men and the 10th in women worldwide. Morbidity and mortality are increasing over the past decade globally despite the improvement in RCC diagnosis and management [1]. The renal clear cell carcinoma (ccRCC) subtype accounts for nearly 70–75% of all primary renal cancers [2]. For non-metastatic ccRCC, radical or partial nephrectomy remains the most effective therapy. However, after nephrectomy, RCC recurs in ca 25% of patients [3]. One-third of ccRCC patients are diagnosed with locally advanced or metastatic disease and have a poor prognosis with a <12% 5-year survival rate [4]. Emerging treatment modalities, such as targeted therapy and immunotherapy have improved survival in these patients, but the effects are still limited [5]. Therefore, further investigation of the mechanism underlying ccRCC pathogenesis is important to identify new diagnostic and therapeutic targets.

Leucine Zipper Transcription Factor-like 1 (LZTFL1) is a tumor suppressor located in the chromosome region 3p21.3 [6]. Deletions of 3p21.3 are a frequent and early event in many cancer types including the kidney cancer [7]. Previously we identified a tumor suppressive function of LZTFL1 in gastric cancer [8] and showed that LZTFL1 can suppress gastric cancer cell migration and invasion through regulating nuclear translocation of β-catenin [9]. LZTFL1 is also known as BBS17 that interacts with a BBS (Bardet-Biedl Syndrome) protein complex known as the BBSome and may act as a cargo protein to regulate ciliary trafficking of the BBSome [10]. Loss-function of *Lztfl1* in *Lztfl1*-knock out mice resulted in pleiotropic phenotypes including obesity, which resemble patients with BBSome [11]. LZTFL1 is also expressed in ciliated human bronchial epithelial cells and inhibits lung tumorigenesis, possibly by maintaining epithelial cell differentiation and/or inhibition of signaling that leads to epithelial-to-mesenchymal transition (EMT) [12]. Recently, there is a renowned interest in biology of *LZTFL1* as it is emerged as a candidate effector gene at a COVID-19 risk locus [13–15].

The role of LZTFL1 in RCC remains unknown. Previously, we identified LZTFL1 as a target of miR-106b-5p that promotes RCC aggressiveness and found that lower LZTFL1 expression was associated with shorter overall survival time (OS) of ccRCC patients through analysis of LZTFL1-mRNA in The Cancer Genome Atlas (TCGA) pan-cancer database [16]. To understand the role of LZTFL1 in kidney tumorigenesis, here we carried out further bioinformatics analysis and experimental validation. Furthermore, we tested the role of LZTFL1 in kidney tumor cell growth both in vitro and in vivo through gain and loss-functional studies. Our data show that LZTFL1 inhibits kidney tumor cell growth both in vitro and in vivo. Mechanistically, we found that LZTFL1 inhibits kidney tumor cell cycle progression and suppresses the AKT signaling by destabilizing AKT via ZNRF1-mediated ubiquitin proteosome pathway (UPP). Finally, we show that overexpression of LZTFL1 in patient-derived xenografts (PDX) inhibited tumor growth. Our studies indicate that LZTFL1 is frequently deleted in ccRCC and low LZTFL1 expression is associated with a poor outcome. Reactivation or re-expression of LZTFL1 in ccRCC may have clinical significance in kidney cancer therapy.

## Results

### LZTFL1 is frequently deleted in ccRCC and low LZTFL1 transcript is associated with a poor ccRCC outcome

**Fig. 1:**
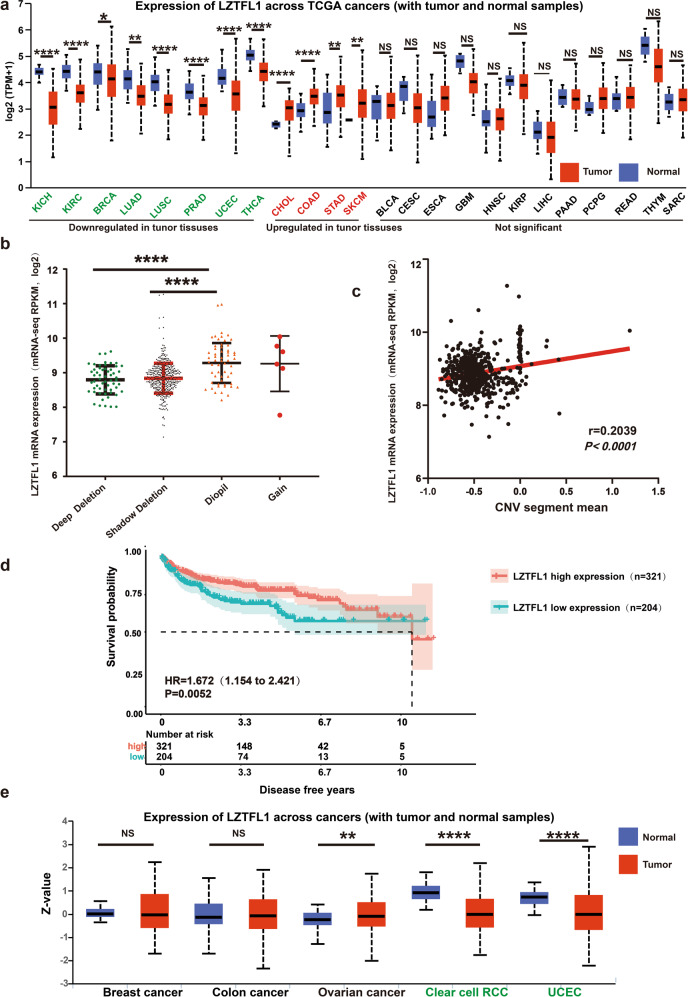
LZTFL1 is significantly down regulated in ccRCC compared to normal tissues. **a** The expression level of LZTFL1 in tumor and normal tissues based on data from the TCGA database. **b** Relative mRNA level as a function of the relative copy number of LZTFL1 in the TCGA database. **c** Correlation of LZTFL1 mRNA and CNV segment means from ccRCC patients using TCGA level 3 data. **d** Kaplan–Meier analysis of survival and the COX proportional hazards model for the hazard ratio of LZTFL1 mRNA levels as a prognostic marker in ccRCC patients. **e** LZTFL1 protein level in ccRCC patient’s tissue samples was compared with those in normal renal tissues in the CPTAC database. *, *P* < *0.05*, **, *P* < *0.01*, ***, *P* < *0.001*, ****, *P* < *0.0001*, NS not significant, unpaired *t*-test.

We evaluated LZTFL1 mRNA expression in TCGA pan-cancer database (Supplementary Table 1) through UALCAN portal (http://ualcan.path.uab.edu/) [17]. LZTFL1 was significantly downregulated in 8 tumor types compared to normal tissues, including kidney clear cell carcinoma (KIRC) and kidney chromophobe (KICH) (Fig. 1a). Upregulation of LZTFL1 in some tumors may suggest non-tumor suppressor-related functions as shown in previous studies [11]. Copy number alterations (CNAs) are important predictive and prognostic biomarkers in human cancer and have been defined as copy number variation (CNVs), including duplication, amplification, deletion, and homozygous deletion, in a specific genomic region in somatic cells [18]. We tested LZTFL1 gene alterations in ccRCC patients using the cBio Cancer Genomics Portal [19] (https://docs.cbioportal.org/user-guide/faq/#what-is-gistic-what-is-rae). Analysis of LZTFL1 CNA distribution showed that only a small number of tumor samples (*n* = 6) have gain of LZTFL1 whereas deep deletion and shallow deletion of LZTFL1 are overrepresented in the TCGA database (*n* = 57 deep deletion and *n* = 406 shallow deletion). No amplification of LZTFL1 was found (Fig. 1b). Tumors with LZTFL1 deep or shallow deletion showed lower LZTFL1 mRNA expression compared to tumors without deletion (diploid) (Fig. 1b). Two-sided Pearson’s correlation study showed that LZTFL1 mRNA expression positively correlated with CNV segment mean [20] in TCGA level 3 dataset (Fig. 1c) and in 29 renal cell carcinoma cell lines in Cancer Cell Line Encyclopedia (CCLE) database (https://sites.broadinstitute.org/ccle/datasets) (Supplementary Fig. 1).

Previously, we found that lower LZTFL1 expression was associated with shorter overall survival time (OS) of ccRCC patients [16]. To test whether LZTFL1 predicts reduced disease-free survival (DFS) time, we conducted X-tile analysis [21] to determine the optimal grouping cut-off points (Supplementary Fig. 2a). Low LZTFL1 expression group showed a worse DFS than high LZTFL1 expression group (Fig. 1d). Furthermore, we examined LZTFL1 expression at protein level in ccRCC samples from CPTAC database [22]. LZTFL1 protein level was found to be significantly decreased in the ccRCC group compared to that in normal group (*n* = 84 Normal, *n* = 110 Primary tumor) (Fig. 1e).

### Downregulation of LZTFL1 in ccRCC patients correlates with poorer survival outcome

**Fig. 2:**
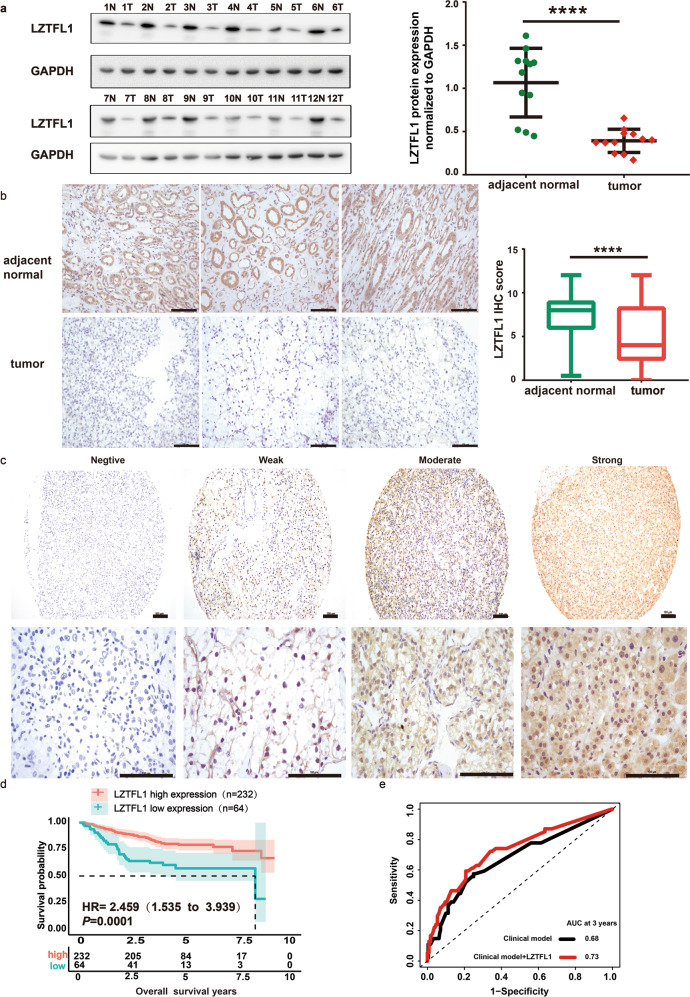
Low expression of LZTFL1 predicts poor OS in ccRCC patients. **a** Left panel, Western blots of LZTFL1 protein level in 12 pairs of matched ccRCC (T) and adjacent normal tissues (N). Right panel, LZTFL1 levels were quantified by densitometry with ImageJ and normalized by GAPDH. Means ± SD, ****, *P* < *0.0001*, paired *t*-test. **b** Left panel, representative images of LZTFL1 immunohistochemical (IHC) staining in tumor and adjacent normal tissues with different pathological characteristics. Right panel, box plots of LZTFL1 IHC staining in adjacent normal tissue (*n* = 266) and recurrent (*n* = 266) patients. ****, *P* < *0.0001*, paired *t*-test, scale bar is 100 μm. **c** Representative images of different LZTFL1 IHC staining scores in ccRCC tissues, scale bar is 100 μm. **d** Kaplan–Meier analysis of overall survival and the COX proportional hazards model for the hazard ratio of LZTFL1 protein levels as a prognostic marker in ccRCC patients. **e** Comparison of the predictive accuracy of outcome by the clinical model alone or with LZTFL1.

To verify the results obtained from public databases and bioinformatic analysis, we analyzed LZTFL1 protein expression in ccRCC samples obtained from our affiliated hospital. LZTFL1 protein expression was decreased in tumors (T) compared to their adjacent normal tissues (N) (Fig. 2a). In order to determine the relationship between the expression of LZTFL1 and the outcomes of ccRCC patients, LZTFL1 expression level was detected by immunohistochemistry (IHC) in a tissue microarray of 296 ccRCC samples (SYSU set). No significant correlations of LZTFL1 expression were found with the age, gender, Fuhrman grade, necrosis, vascular invasion, or TNM stage (*P* > 0.05, Table 1). While adjacent specimens exhibited remarkably high expression of LZTFL1 in the cytoplasm, the matched ccRCC samples consistently showed weaker or undetectable immunostaining of LZTFL1 (Fig. 2b). The LZTFL1 expressions in the IHC staining of ccRCC tissue microarray were scored (Fig. 2c). 64 and 232 patients in the cohort were separated into low and high LZTFL1 expression groups, respectively, using X-tile plots to generate the optimum cut-off score (Supplementary Fig. 2b). The overall survival was significantly better for patients with tumors showing moderate or strong LZTFL1 expression (IHC score > 2) than for those whose tumors showed negligible or weak expression (IHC score ≤ 2; median survival time: 8.33 years, *P* = 0.0001, log-rank test; Fig. 2d). Although the median survival was not achieved due to insufficient follow-up time for patients with an IHC score of >2, patients in this group did show lower risk of death with hazard ratio of 2.459 (95% confidence interval,1.535–3.939).

**Table 1:** Correlation expression of LZTFL1 and clinicopathological variables in 296 cases of ccRCC.

*Variable*	*All cases* (*N* = 296)	*LZTFL1 expression* (%)		*P value*^a^
		*Low expression* (*N* = 64)	*high expression* (*N* = 232)	
Age(years)				0.578
≤55	164 (55.4)	33 (51.6)	131 (56.5)	
>56	132 (44.6)	31 (48.4)	101 (43.5)	
Gender				0.853
Male	204 (68.9)	43 (67.2)	161 (69.4)	
Female	92 (31.1)	21 (32.8)	71 (30.6)	
Fuhrman nuclear grade				0.685
1	45 (15.2)	7 (10.9)	38 (16.4)	
2	174 (58.8)	38 (59.4)	136 (58.6)	
3	59 (19.9)	15 (23.4)	44 (19.0)	
4	18 (6.1)	4 (6.2)	14 (6.0)	
Necrosis				0.983
No	220 (74.3)	47 (73.4)	173 (74.6)	
Yes	76 (25.7)	17 (26.6)	59 (25.4)	
Vascular invasion				0.288
No	271 (91.6)	56 (87.5)	215 (92.7)	
Yes	25 (8.4)	8 (12.5)	17 (7.3)	
Lymph nodes invasion				>0.9
No	275 (92.9)	59 (92.2)	216 (93.1)	
Yes	21 (7.1)	5 (7.8)	16 (6.9)	
TNM stage				0.318
I	195 (65.9)	39 (60.9)	156 (67.2)	
II	64 (21.6)	17 (26.6)	47 (20.3)	
III	30 (10.1)	5 (7.8)	25 (10.8)	
IV	7 (2.4)	3 (4.7)	4 (1.7)	

^a^Chi-square test.

We instigated the prognostic value of LZTFL1 and compared it with other prognostic factors of ccRCC, including age, Fuhrman grade, tumor necrosis, lymph node invasion, and TNM stage. Similar to Fuhrman grade and lymph node invasion, the IHC score of LZTFL1 expression significantly predicted OS rate in both univariate and multivariate analysis (Table 2), suggesting that LZTFL1 expression may be used as an independent prognostic factor of ccRCC. Moreover, the predictive ability of the prognostic model was slightly improved by the inclusion of LZTFL1 expression level, as demonstrated by the increase of the resulting area under the curve (AUC) value from 0.68 to 0.73 at the 3rd year of follow up (Fig. 2e). Taken together, these results suggest that LZTFL1 downregulation predicts poor OS in ccRCC and the expression of LZTFL1 might add prognostic value to the staging and grading system of ccRCC.

**Table 2:** Univariate and multivariate analysis of prognostic parameters in 296 cases of ccRCC.

*Variable*	*All cases* (%)	*Univariate*^*a*^		*Multivariate*^*b*^	
		*HR (95% CI)*	*P value*	*HR (95% CI)*	*P value*
Age(years)			**0.006**		**0.0273**
≤55	164 (55.4)	1		1	
>56	132 (44.6)	1.899 (1.2–3.004)		1.6870 (1.0603–2.6839)	
Gender			0.743		
Male	204 (68.9)	1			
Female	92 (31.1)	1.084 (0.6707–1.751)			
Fuhrman nuclear grade			**<0.0001**		**0.00659**
1–2	219 (74)	1		1	
3–4	77 (26)	2.783 (1.768–4.379)		2.0648 (1.2238–3.4837)	
Necrosis			**<0.0001**		0.19359
No	220 (74.3)	1		1	
Yes	76 (25.7)	2.534 (1.606–3.998)		1.4376 (0.8317–2.485)	
Vascular invasion			**0.0389**		0.87578
No	271 (91.6)	1		1	
Yes	25 (8.4)	2.02 (1.036–3.935)		0.9434 (0.4546–1.9580)	
Lymph nodes invasion			**<0.0001**		**0.00518**
No	275 (92.9)	1		1	
Yes	21 (7.1)	4.216 (2.354–7.552)		2.4738 (1.3109–4.668)	
TNM stage			**<0.0001**		0.0576
I–II	259 (87.5)	1		1	
III–IV	37 (12.5)	2.783 (1.654–4.681)		1.7390 (0.9823–3.0784)	
LZTFL1 IHC score			**<0.0001**		**<0.0001**
>2	232 (78.4)	1		1	
≤2	64 (21.6)	2.459 (1.535–3.939)		2.076 (1.67–4.384)	

*HR* hazards ratio, *CI* confidence interval.

^a^Log-rank test.

^b^Cox regression model.

Bold values identify statistical significance (*P* < 0.05)

### LZTFL1 inhibits ccRCC cell growth and proliferation in vitro and in vivo

**Fig. 3:**
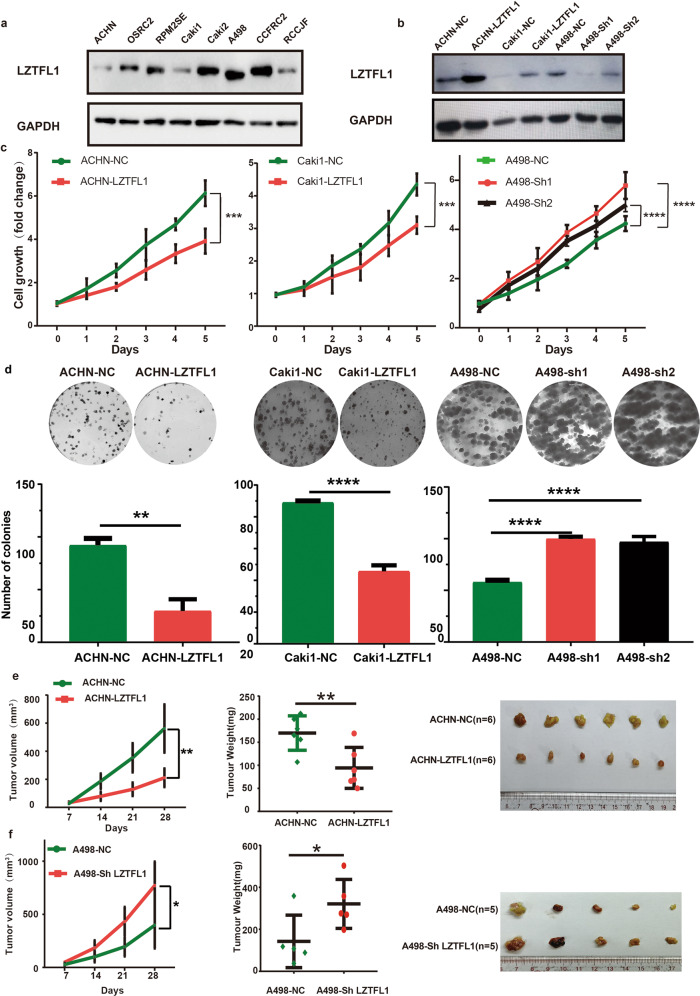
LZTFL1 suppresses cell proliferation in vitro and in vivo. **a** Western blots of the endogenous LZTFL1 in various ccRCC cell lines. GAPDH was used as loading control. **b** Western blots of LZTFL1 in ACHN and Caki1 cell lines transduced with lentiviruses expressing control vector (ACHN-NC, caki1-NC) or LZTFL1 (ACHN-LZTFL1, Caki1-LZTFL1), and in A498 cell line transduced with lentiviruses expressing control (A498-NC) or two different LZTFL1 shRNAs (A498-sh1 & A498-sh2). **c** Relative cell growth of ccRCC cell lines with control, LZTFL1-overexpressed or knocked down as indicated. Mean ± SD, ****, *P* < 0.0001, two-way ANOVA. **d** Colony forming ability of ccRCC cells with control, LZTFL1-overexpressed or knocked down as indicated. *N* = 3, mean ± SD, **, *P* < *0.01*, ****, *P* < 0.0001, Student’s *t* test. **e**, **f** 5 × 10^6^ cells with LZTFL1 overexpressed (**e**), knockdown (**f**), or corresponding control vectors were inoculated subcutaneously into the mice. Tumor volume was recorded weekly (left panel). Data are shown as mean ± SD, *, *P* < 0.05, ***, *P* < 0.001, two-way ANOVA. Tumor weight (middle panel) and tumor micrographs (right panel) at time of sacrifice were shown. mean ± SD, *, *P* < *0.05*, **, *P* < *0.01*, Student’s *t* test.

To investigate the pathophysiological role of LZTFL1 in ccRCC, we analyzed LZTFL1 expression in various established ccRCC cell lines. Among eight ccRCC cell lines we tested, LZTFL1 expression is downregulated in ACHN, Caki1, and RCCJF (Fig. 3a). We re-expressed LZTFL1 stably in low-LZTFL1 expressing ACHN and Caik1 cell lines and knocked down LZTFL1 in high-LZTFL1 expressing A498 cell line (Fig. 3b). Proliferation assays by Cell Counting Kit-8 (CCK-8) and colony formation assays revealed that over-expression of LZTFL1 in ACHN and Caki1 cells inhibited cell growth and proliferation capacity. Conversely, knockdown of LZTFL1 in A498 cells enhanced cell growth and colony formation ability significantly (Fig. 3c, d). Overexpression of LZTFL1 also reduced the size and weight of subcutaneous xenografts in vivo (Fig. 3e). Conversely, knockdown of LZTFL1 promoted the growth of A498 xenograft in vivo (Fig. 3f).

### LZTFL1 inhibited cell proliferation by blocking the cell cycle progression

**Fig. 4:**
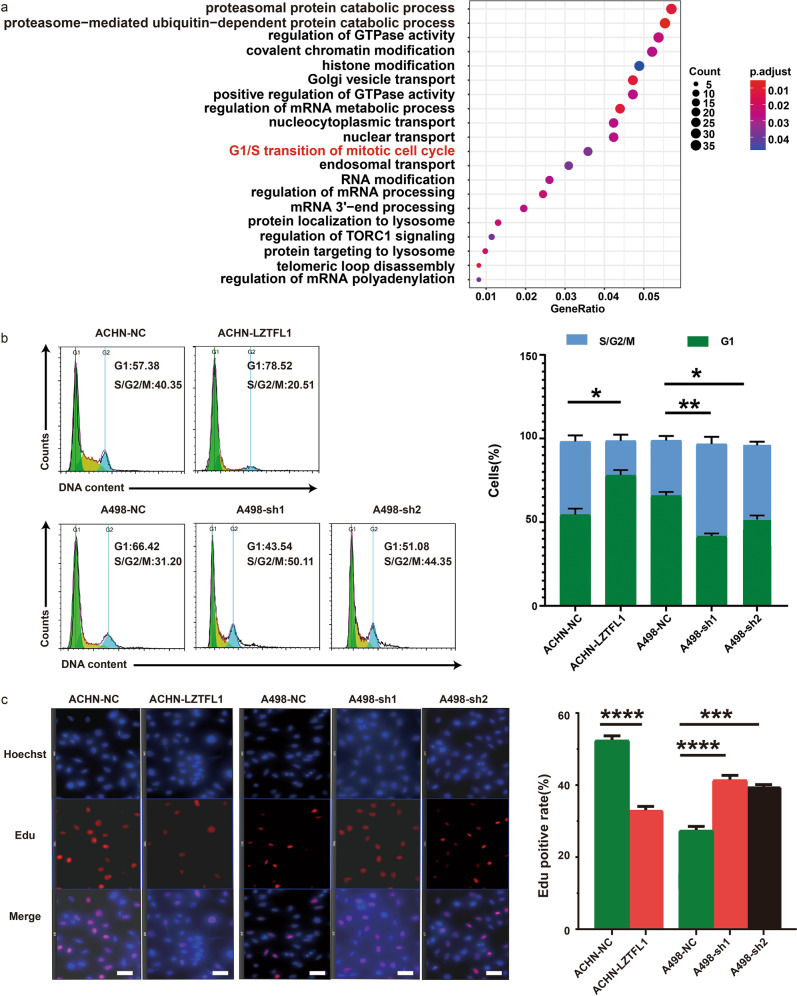
LZTFL1 induces cell cycle arrest at G1/S transition. **a** GO terms of differentially expressed genes (DEGs) between lower and upper quartile expression of LZTFL1 in TCGA-ccRCC cohort. Count indicates the number of DEGs enriched in the pathway. GeneRatio indicates the ratio of enriched DEGs to background genes. **b** Flow cytometry cell cycle analysis of ACHN and A498 cells stably overexpressing LZTFL1 and knockdown, respectively, and their respective control cells (left panels). Percentages (%) of cell populations at different cell cycle phases (right panels). mean ± SD of three independent experiments, *, *P* < *0.05*, **, *P* < *0.01*, unpaired student *t* test. **c** Representative immunofluorescent staining of Edu (red) and DAPI (blue) of cells indicated (left panel) and % of Edu-positive cells (right panel). Mean ± SD of three independent experiments, ***, *P* < *0.001*, ****, *P* < *0.0001*, unpaired student *t* test. scale bar is 60 μm.

To understand the mechanism by which LZTFL1 inhibits ccRCC tumorigenesis, we performed differential gene expression analysis using TCGA-ccRCC cohort dataset. We first identified low and high-LZTFL1 expression groups based on their LZTFL1 mRNA in the lower and upper quartile level in the dataset, respectively. We then used a published protocol [23] and identified 682 differentially expressed gene (DEGs) between these two groups (Supplementary Fig. 3). Gene ontology (GO) analyses of these DEGs revealed a total of 20 GO terms under the threshold of *P* < *0.05*, including G1/S transition of the cell cycle (Fig. 4a, highlighted in red). We tested whether LZTFL1 influenced the cell progression using flow cytometry and Edu cooperation assays. As shown in Fig. 4b, overexpression of LZTFL1 led to cell cycle arrest at G1/S while knockdown of LZTFL1 facilitated cell cycle transition from G1 to S (Fig. 4b). Edu cooperation assays also indicated that LZTFL1 overexpression downregulated the population of Edu-positive cells in ACHN-LZTFL1 cells compared to ACHN-NC cells. Conversely, LZTFL1 knockdown in A498 cells (A498-sh1, A498-sh2) increased the fraction of Edu-positive cells compared to A498 cells with nonspecific knockdown (A498-NC) (Fig. 4c).

### LZTFL1 interacts with ZNRF1 that downregulates the expression of AKT by ubiquitination and degradation

**Fig. 5:**
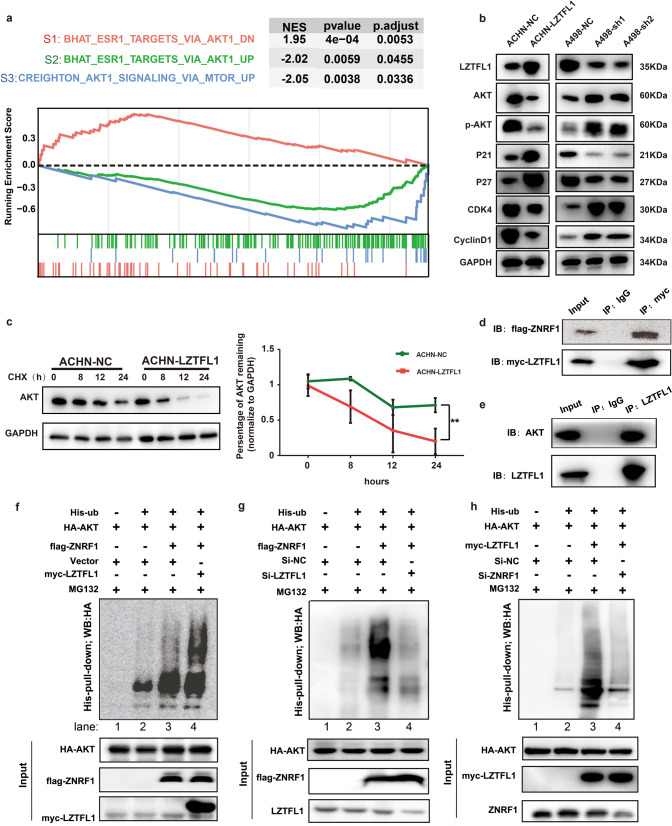
LZTFL1 destabilizes AKT through ZNRF1-mediated ubiquitin proteosome degradation pathway. **a** GSEA of genes in TGCA cohorts that express high and low LZTFL1, respectively. **b** Western blot of proteins indicated in ACHN-NC, ACHN-LZTFL1, A498-NC, A498-sh1, andA498-sh2 cells. **c** Western blot of LZTFL1 in ACHN-NC and ACHN-LZTFL1 cells at various time points after addition of translational inhibitor cycloheximide (CHX) (left panel). The expression levels of LZTFL1 in Western blots were quantified by densitometry and normalized against loading control GAPDH (right panel). Mean ± SD of three independent experiments. *, *P* < *0.05*, two-way ANOVA. **d** HEK293T cells transfected with myc-LZTFL1 and Flag-ZNRF1 were immunoprecipitated with control IgG or anti-myc antibody and western blotted with anti-flag or anti-myc antibody. 10% of input was loaded on the gel. **e** HEK293T cells were immunoprecipitated with control IgG or anti-LZTFL1 antibody and western blotted with anti-AKT or anti-LZTFL1 antibody. 10% of input was loaded on the gel. **f**–**h** HEK293T cells (**f**) and A498 cells (**g**, **h**) transfected with plasmids expressing indicated proteins were treated with MG132 for 4 h before harvested. His-ubiquitin (His-ub) conjugated proteins were pulled down with Ni-NTA agarose beads and subjected to Western blot analysis with anti-HA antibody.

To further understand the molecular mechanism by which LZTFL1 regulates the cell cycle progression in ccRCC cells, Gene set enrichment analysis (GSEA) [24] was performed to identify gene sets that correlate with LZTFL1 expression in TGCA cohorts that express high and low LZTFL1 (Supplementary Fig. 3), respectively. Genes are ranked based on the fold change between these two groups. Three gene sets were identified by GSEA with absolute normalized enrichment score |NES | > 1.5 (Fig. 5a); S1 (BHAT_ESR1_TARGETS_VIA_AKT1_DN), S2 (BHAT_ESR1_TARGETS_VIA_AKT1_UP), and S3 (CREIGHTON_AKT1_SIGNALING_VIA_MTOR_UP). S1 contains genes bound by ESR1 and down-regulated by estradiol in MCF-7 cells expressing constitutively active form of AKT1. Gene set S1 has a positive enrichment score (ES) peak and is correlated with LZTFL1 expression (red curve), suggesting genes repressed in the AKT pathway are highly enriched in the high LZTFL1 expressing samples. Gene sets S2 and S3 contain genes that are upregulated by AKT/MTOR and have negative ES peak that is inversely correlated with LZTFL1 expression, suggesting genes induced by AKT are enriched in low-LZTFL1 expressing samples. These data suggest that genes in the AKT/mTOR signaling pathway were negatively regulated by LZTFL1. We confirmed this by western blot analysis, showing LZTFL1 overexpression in ACHN cells downregulated the expression of AKT, CDK4, and cyclinD1 and upregulated the expression of cell cycle inhibitors P21 and P27 (Fig. 5b, left panel). Conversely, LZTFL1 knockdown in A498 cells had opposite effect on the expression of these proteins (Fig. 5b, right panel).

As LZTFL1 did not impact the transcription of AKT in kidney tumor cells (Supplementary Fig. 4) and the GO suggested that LZTFL1 may be involved in proteasomal protein catabolic process and ubiquitination (Fig. 4a), we tested whether LZTFL1 regulates the stability of AKT. The cycloheximide (CHX) chase experiment showed that the half-life of the AKT protein in ACHN-LZTFL1 cells was significantly decreased compared with that in control ACHN-NC cells (Fig. 5c). To understand how LZTFL1 may affect AKT protein stability, we searched the Biological General Repository for Interaction Datasets (BioGRID) to identify potential LZTFL1-interactive proteins [25]. There are 40 proteins listed in the BioGRID database that potentially interact with LZTFL1. GO analyses of these proteins revealed 136 GO terms under the threshold of *P* < 0.05, including three enriched GO terms in proteasomal protein catabolic process or ubiquitination. E3 ubiquitin-protein ligase ZNRF1 is one of the proteins involved in the LZTFL1-interactive network. ZNRF1 was shown to target AKT for proteasomal degradation [26, 27]. We confirmed that LZTFL1 interacted with ZNRF1 (Fig. 5d) and AKT (Fig. 5e) in co-immunoprecipitation assays. LZTFL1 also upregulated colocalization of AKT and ZNRF1 (Supplementary Fig. 5). LZTFL1 had no effect on the transcription of ZNRF1 (Supplementary Fig. 4). We performed ubiquitination assay by co-transfecting HEK293T cells with or without myc-LZTFL1, flag-ZNRF1, HA-AKT, and His-ubiquitin (His-ub). AKT is ubiquitylated (Fig. 5f, lane 2 vs 1). The level of polyubiquitylated AKT was increased in the presence of ZNRF1 (Fig. 5f, lane 3) and further upregulated by LZTFL1 (Fig. 5f, lane 4). Conversely, the amount of ubiquitination of AKT was decreased in A498 cells with LZTFL1 knockdown (Fig. 5g, lane 4 vs 3). ZNRF1 knockdown partially abolished the increased AKT ubiquitination induced by LZTFL1 overexpression (Fig. 5h).

### ZNRF1 knockdown partially rescued the cell growth potential suppressed by LZTFL1

**Fig. 6:**
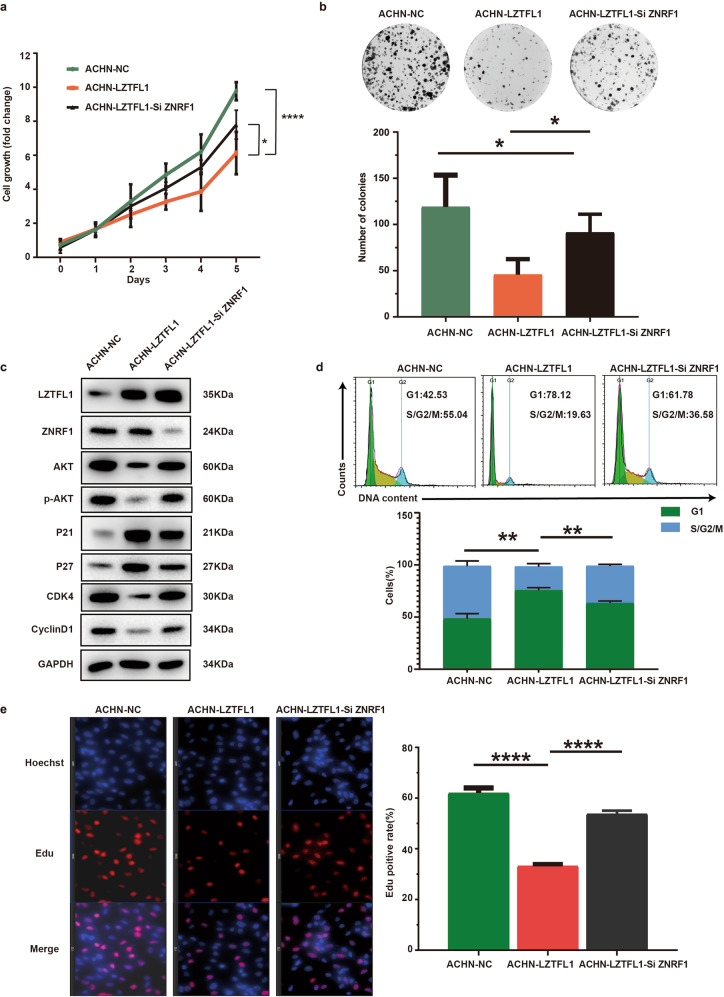
ZNRF1 knockdown partially rescued cell proliferation inhibited by LZTFL1. **a** Relative cell growth of ACHN-NC, ACHN-LZTFL1, and ACHN-LZTFL1-siZNRF1 cells. Mean ± SD of three independent experiments, *, *P* < 0.05, ***, *P* < 0.001, two-way ANOVA. **b** Colony forming ability of ACHN-NC, ACHN-LZTFL1 and ACHN-LZTFL1-siZNRF1. **a**, **b** Mean ± SD of three independent experiments, *, *P* < *0.05*, ****, *P* < *0.0001*, student’s *t* test. **c** Western blots of proteins indicated in ACHN-NC, ACHN-LZTFL1 and ACHN-LZTFL1-siZNRF1 cells. **d** FACS of ACHN-NC, ACHN-LZTFL1 and ACHN-LZTFL1-siZNRF1 (top panel) and % cell populations at different stages of cell cycles (bottom panel). Mean ± SD of three independent experiments, **, *P* < *0.01*, unpaired student *t* test. **e** Representative immunofluorescence micrographs stained with Edu (red) and DAPI (blue) of ACHN-NC, ACHN-LZTFL1 and ACHN-LZTFL1-siZNRF1 cells (left panel). % Edu-positive cells from the left panel were quantified (right panel). Mean ± SD of three independent experiments, ****, *P* < *0.0001*, unpaired student *t* test.

We tested the effect of ZNRF1 knockdown on LZTFL1-regulated cell proliferation and AKT signaling. ZNRF1 was knocked down in ACHN-LZTFL1 cells. Proliferation assays by CCK-8 and colony formation assays revealed that over-expression of LZTFL1 in ACHN cells inhibited cell proliferation whereas ZNRF1 knockdown partially reversed the effect of LZTFL1 (Fig. 6a and b). Overexpression of LZTFL1 in ACHN cells also downregulated the protein levels of AKT, CDK4, and CyclinD1 and upregulated the protein levels of P21 and P27, which are reversed by ZNRF1 knockdown (Fig. 6c). Moreover, ZNRF1 knockdown significantly reversed the G1 cell cycle arrest caused by LZTFL1 over-expression (Fig. 6d). ZNRF1 knockdown also upregulated Edu-labeled cells that was downregulated by LZTFL1-overexpression (Fig. 6e). Taken together, these results suggest that the effect of LZTFL1 on the AKT protein stability is mediated through ZNRF1, which is at least partially responsible for the anti-cell proliferative function of LZTFL1 on kidney tumor cells.

Previously we found that LZTFL1 can suppress gastric cancer metastasis by regulating β-catenin signal and suppress lung tumorigenesis, possibly affecting epithelial cell identity and/or EMT [9, 12]. We performed qRT-PCR analysis of genes involved in the β−catenin and EMT signaling pathways in stable cell lines that overexpress or knockdown LZTFL1, respectively. None of the genes had more than 2-fold change of gene expression (Supplementary Fig. 6). We also performed transwell assays with stable cell lines that overexpress LZTFL1 to test whether LZTFL1 promotes cell migration. Over-expression of LZTFL1 had no significant effect on invasion capacity (Supplementary Fig. 7).

### LZTFL1 inhibits ccRCC growth in a PDX model

**Fig. 7:**
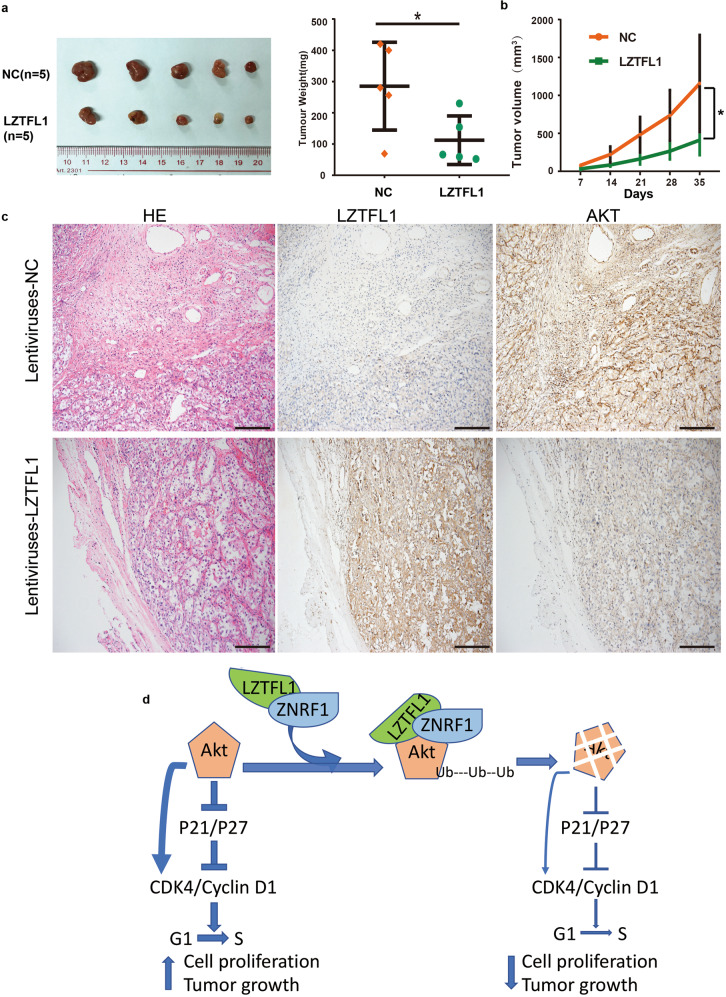
LZFL1 inhibits ccRCC growth in patient-derived xenograft (PDX) model. **a** Tumor micrograph (left panel) and weight (right panel) at time of sacrifice from PDXs treated with lentiviruses expressing control vector (NC) or LZTFL1. Mean ± SD, *, *P* < *0.05*, unpaired *t*-test. **b** Tumor volume during treatment. Mean ± SEM, *, *P* < *0.05*, two-way ANOVA. **c** H&E and IHC staining of PDX tumors, scale bar is 100 μm. **d** Schematic diagram of the role of LZTFL1 in ccRCC. LZTFL1 binds to ZNRF1 and AKT, promotes AKT degradation through the UPP pathway, leading to cell cycle arrest and inhibition of ccRCC growth.

PDX models specifically reflect the patient’s tumor heterogeneity and diversity, providing an essential oncology research platform on which to investigate the molecular mechanisms of tumor growth and predict the response to anticancer treatment [28–30]. We established a PDX model with fresh ccRCC tissue and evaluated the effect of LZTFL1-targeted cancer therapy via intra-tumoral injection of lentiviruses expressing LZTFL1 (twice weekly, for a total of 10 times). The lentiviruses were efficiently taken up by the tumor as assayed by immunofluorescent staining of GFP encoded by the GFP reporter gene from lentiviral vector (Supplementary Fig. 8). LZTFL1 overexpression significantly suppressed tumor growth in PDX mouse model compared to lentiviruses expressing control vector (Fig. 7a and b). No side effects of lentiviral-delivery of LZTFL1 were observed on tumor-bearing mice (Supplementary Fig. 9). H&E staining confirmed the pathological type of the tumors harvested from PDX model as renal clear cell carcinoma and IHC analysis revealed that AKT expression was decreased in the LZTFL1 treated group (Fig. 7c). These findings from PDX model suggest that LZTFL1 overexpression inhibits tumor growth and may have clinical significance in the future.

## Discussion

In this work, we hypothesized a tumor suppressive function of LZTFL1 in ccRCC and its mechanism of action based on extensive bioinformatics analysis of public patients’ tumor data and validated it using both LZTFL1 gain- and loss-functional studies in kidney tumor cell lines and PDX model systems. Our studies showed that LZTFL1 inhibits kidney tumor cell growth and cell cycle G1 to S phase transition. Clinically, we found that LZTFL1 is frequently deleted in ccRCC. Downregulation of LZTFL1 is associated with a poor outcome and may be used as a prognostic marker for ccRCC.

The effect of LZTFL1 on tumor cell growth is likely due to its inhibition on AKT. We found that LZTFL1 inhibited G1 to S phase cell cycle progression in kidney tumor cells. In general, cell cycle is primarily regulated by cyclins, cyclin-dependent kinases (CDKs), and CDK inhibitors such as P21 and P27. AKT is known to promote cell growth and proliferation by inhibiting P21 and P27 and regulating the activities of Cyclin D1 and CDK4 at G1/S cell cycle checkpoint [31–35]. P21 and P27 bind CDKs, inhibiting enzymatic activities of CDKs and arresting cell cycle at G1 phase. AKT phosphorylates P21 and P27, releasing their inhibition on CDK4 [36–39]. AKT can also promote CDK4 activity via non-P21/P27 dependent pathways [34]. Downregulation of AKT could result in upregulation of P21 and P27 and downregulation of CDK4, resulting in inhibition of cell cycle progression (Fig. 7d). Consistently, we found that LZTFL1-overexpression downregulated AKT (Fig. 5) and upregulated P21 and P27 whereas LZTFL1 knockdown had opposite effects. Together these data suggest that LZTFL1 may inhibit G1 to S phase cell cycle transition via the AKT signaling pathway.

The majority of intracellular proteins are degraded by the ubiquitin proteasome pathway (UPP) [40, 41]. Targeted proteins are polyubiquitinated by the protein complex that includes activating enzyme E1, ubiquitin conjugating enzyme E2, and the ubiquitin ligase E3, which is then recognized and subsequently degraded by the 26 S proteasome. UPP plays an important role in cell cycle progression by regulating the turnover of proteins involved in cell cycle control [42]. AKT degradation by E3 ubiquitin ligase ZRNF1 has been shown to promote neuronal degeneration [27, 43]. Our studies showed that ZNRF1 also targets AKT for degradation in kidney tumor cells. AKT is polyubiquitinated in the kidney tumor cells in the presence of ZNRF1 (Fig. 5f). Polyubiquitination of AKT is upregulated in LZTFL1 overexpressing cells (Fig. 5f) and downregulated in LZTFL1 knockdown cells (Fig. 5g). Upregulation of AKT polyubiquitylation by LZTFL1 is ZNRF1-dependent as knockdown of ZNRF1 abolished LZTFL1-upregulated AKT polyubiquitination (Fig. 5h). LZTFL1 interacted with both ZNRF1 and AKT (Fig. 5d,e) and upregulated colocalization of AKT and ZNRF1 (Supplementary Fig. 5). LZTFL1 did not impact the transcription of AKT and ZNRF1 (Supplementary Fig. 4). Together, these data suggest the following mechanistic model: LZTFL1 acts as a scaffold that brings AKT and ZNRF1 to each other and increases polyubiquitylation of AKT, leading to AKT degradation through ZNRF1-mediated UPP pathway. Since AKT is known to promoter tumor cell proliferation/tumor growth via inhibition of P21/P27 and upregulation of CDKs/cyclin D1, destabilization of AKT by LZTFL1 could downregulate the signaling in the AKT-P21/P27-CDK4/cyclin D1 axis, leading to reduced cell cycle progression and tumor cell growth (Fig. 7d). That being said, other mechanisms by which LZTFL1 inhibits kidney tumor cell proliferation may also be important as knockdown of ZNRF1 only partially reversed the effect of LZTFL1 on tumor cell growth.

In conclusion, we have identified a novel tumor suppressive function of LZTFL1 in ccRCC. LZTFL1 inhibits kidney tumor cell growth and proliferation. Mechanistically, we found that LZTFL1 may inhibit G1 to S phase cell cycle transition by destabilizing AKT through ZNRF1-mediated UPP. Furthermore, clinically, we found that downregulation of LZTFL1 in ccRCC may correlate with worse outcome and may be used as a prognostic maker; and overexpression of LZTFL1 in kidney tumors may be a therapeutic strategy against ccRCC.

## Materials and methods

### Online patient cohorts and bioinformatics analysis

Clinical information, copy number variant and mRNA data of KIRC samples were obtained from TCGA (https://portal.gdc.cancer.gov) and cBioPortal databases (https://www.cbioportal.org). The dataset consisted of 72 normal controls and 533 KIRC samples. Samples with the lower and upper quartile of LZTFL1 mRNA level were selected as the low and high expression group, respectively, and were used to identify DEGs with the criteria of FDR < 0.01 and |log_2_ Fold Change | ≥1 using the “Limma” R package. GO analyses of DEGs were carried out with the “clusterProfiler” package [44]. GSEA was performed by “clusterProfiler” R package to determine whether prior defined functional or pathway sets of genes differ significantly between high- and low-expression groups [24]. We searched the Molecular Signatures Database on GSEA website (http://www.gsea-msigdb.org/gsea/msigdb/index.jsp), using key words “cell cycle” and “AKT”. Enrichments of gene sets with an *P* value < 0.05, |NES | > 1.5, and FDR < 0.05 were regarded to be significant. All statistical analyses were performed using R software (Version 4.1.1) and R studio (Version: 1.4.1717). The protein expression data was obtained from CTPAC (Clinical Proteomic Tumor Analysis Consortium) database through UALCAN (http://ualcan.path.uab.edu/analysis-prot.html), consisting of 110 primary tumors and 84 normal control samples.

### ccRCC patient samples, tissue microarray, immunohistochemistry (IHC) and IHC evaluation

Tumors from 296 patients who had undergone surgery for localized ccRCC were collected from Sun Yat-sen University (SYSU) between 2004 and 2012. Human tissue samples and clinical data were obtained with written informed consent and the study was approved by the clinical ethics committee of SYSU. Following criteria were used for selection: histologically confirmed primary ccRCC, no neoadjuvant treatment before operation; underwent complete resection, no other malignant tumors, and availability of detailed clinical data including overall survival (OS). TNM stage was conducted according to the American Joint Committee on Cancer (AJCC) TNM Staging System for Kidney Cancer (7th ed. 2010). This study was approved by the Ethics Committee of Sun Yat-Sen University Cancer Center (NO. GZJZ-SB2016-020) and the First Affiliated Hospital of Sun Yat-Sen University (NO. 2022-932). The assay was conducted in accordance with the Declaration of Helsinki.

Tumors were sectioned and arrayed on slides. IHC staining was performed using a published protocol [12] and anti-LZTFL1 antibody (Proteintech,17073-1AP, rabbit polyclonal, 1:200). LZTFL1 immunoreactivity was assessed with a semi-quantitative scoring method, in which both staining intensity and positive areas were recorded. A staining index (values 0–12), obtained as the intensity of LZTFL1 positive staining (negative = 0, weak = 1, moderate = 2, or strong = 3 scores) multiplies the proportion of immune-positive cells of interest (<25% = 1, 25–50% = 2, >50% to <75% = 3, ≥75% = 4 scores) were calculated. LZTFL1 immunoreactivity was divided into low expression (cases with score 0–2) and high expression (cases with scores 3–12) by X-tile analysis.

### Lentiviruses, siRNAs and shRNA, plasmids, transfection, cell lines and culture conditions

Short hairpin RNA (shRNA) lentiviruses directed against LZTFL1 were purchased from the GenePharma (cat# 160711CZ and 16-1167Z, Shanghai, China). For LZTFL1 overexpression lentivirus, cDNA of LZTFL1 was synthesized and cloned into the lentiviral vector that contains a GFP-reporter gene (catalog #16-06792, GenePharma, Shanghai, China). HEK293T cells were transfected with LZTFL1 overexpression plasmid using Lenti-Pac HIV package kit (GeneCopoeia, MD, USA). Supernatants containing lentivirus were collected 48 h after the transfection and concentrated by Lenti-Pac™ lentivirus concentration solution kit (GeneCopoeia Inc., USA). The titer of a lentivirus vector is calculated using limiting dilution method according to the protocol [45]. Infected cells were incubated with 2 μg/ml puromycin for 2 weeks to select stably transfected cells. siRNAs targeting ZNRF1 and scrambled control siRNA were synthesized by RiboBio (Guangzhou, China). siRNAs targeting LZTFL1 and scrambled control siRNA were synthesized by Tsingke (Biotechnology Co., Ltd). siRNA duplexes transfections were carried out using Lipofectamine RNA iMAX Reagent (Invitrogen). The siRNA and shRNA sequences were listed in Supplementary Table 2. Plasmids expressing HA-AKT, His-ubiquitin, and flag-ZNRF1 were purchased from PPL (Nanjing, China). pcDNA-myc-LZTFL1 was described previously [8]. Transfections were performed using Lipofectamine 3000 according to the manufacturer’s protocol (Invitrogen, CA, USA).

ACHN, A498, CAKI-1, CAKI-2, OSRC2, and HEK293T were purchased from ATCC (American Type Culture Collection). CCFRC2, RPM2SE, RCCJF were kindly provided by Dr. Wei Chen (Department of Urology, The First Affiliated Hospital of Sun Yat-sen University, Guangzhou, People’s Republic of China). OSRC2, CCFRC2 and RCCJF were cultured in RPMI-1640 medium (Gibco, China) supplemented with 10% FBS (PAN-Seratech, Germany). ACHN, RPM2SE, HEK293T and A498 were cultured in DMEM (Gibco, China) supplemented with 10% FBS. Caki1 and Caki2 were maintained in McCoy’s 5 A medium with 10% FBS. Cells were cultured at 37 °C with 5% CO_2_ and routinely checked for mycoplasma infection (Beyotime, China). All cell lines were authenticated by the short tandem repeat DNA profiling test and tested negative for mycoplasma contamination.

### CCK8 proliferation and colony formation assays

Cellular growth was measured using the CCK8 proliferation assay kit (HY-K0301, MCE) according to the manufacturer’s protocol. A total of 1500 cells were seeded per well in a 96-well plate. For colony formation assays, a total of 1000 cells were seeded per well in a 6-well plate and cultured for 2 weeks. The colonies were fixed with 4% paraformaldehyde for 20 min at room temperature and then stained with 0.1% crystal violet. The number of colonies (>50 cells) was counted using ImageJ software.

### Co-immunoprecipitation, western blot, and antibodies

Co-immunoprecipitation and western blot analysis were performed using standard protocols. The following antibodies were used: LZTFL1 (17073-1AP, 1:2000), GAPDH (60004-1-IG, 1:2000), His-tag (66005-1-Ig, 1:2000), HA-tag (51064-2-AP, 1:2000), and myc tag (16286-1-AP, 1:2000) (Proteintech, China); cell cycle regulation antibody sampler Kit (#9932, 1:1000), AKT (#9272, 1:1000) and pAKT(#9271, 1:1,000) (Cell Signaling); ZNRF1 (ABP60990, 1:1000) (Abbine); Flag-tag (F7425,1:2000) (Sigma-Aldrich). The signals of the antigen–antibody complexes were detected by an enhanced chemiluminescence.

### Flowcytometry cell cycle analysis and Edu cell proliferation assay

Cells were seeded in 10 cm dishes. The culture medium was changed to serum free medium for 24 h to facilitate cell cycle synchronization. The cells were collected, washed with PBS and fixed overnight with cold 70% clod ethanol at 4 °C. The fixed cells were analyzed with flow cytometry (cytoFLEX, Beckman,USA) using the Cell Cycle Analysis Kit (4 A biotech, Beijing, China) following the manufacturer’s instructions. The percentage of cells at different stages of cell cycle was calculated using NovoExpress version 1.1.1. Cells transfected with control or various expression plasmids/siRNAs were harvested and reseeded in 48-well plates for Edu assays. The Edu assay kit (RiBoBio, China) was used to determine the proliferation rate of cells according to the manufacturer^’^s instructions.

### Ubiquitination assay

Cells were transfected with control vector plasmid or with myc-LZTFL1, flag-ZNRF1, AKT–HA and His-ubiquitin. 48 h after transfection, cells were incubated with 20 µM MG132 for 4 h. The medium was aspirated, and 1 ml of PBS was added. Cells were scraped off and centrifuged at 2500 *rpm* for 3 min and resuspended by buffer A (6 M Guanidine-HCl, 0.1 M Na_2_ HPO_4_, 0.1 M NaH_2_PO_4_, 10 mM imidazole, pH 8.0). After being sonicated and centrifuged, cell lysates were incubated with 50 µl Ni-NTA agarose beads (QIAGEN, MD, USA) for 4 h at room temperature. The pull-down products were washed once with buffer A, once with 1:3 buffer A/buffer TI (25 mM Tris-HCl, 20 mM imidazole, pH 6.8), and twice with buffer TI. The His-Ub conjugated proteins pulled down by the bead were analyzed by Western blotting.

### Xenograft and PDX studies

Animal care and experiments were conducted with the approval of the Institutional Animal Care and Use Committee of SYSU according to established guidelines. All animal research programs were approved by the animal ethics committee of SYSU. For xenograft mouse model, 5 × 10^6^ cells were injected subcutaneously into the flanks of 4-week-old BALB/c nude mice. The xenograft volume was measured weekly using the formula: V = 0.5 × length × width^2^. Mice were sacrificed after 7 weeks, and tumors were excised. The tumor weight was measured and subjected to histological examination. Commercial NOD/ShiLtJGpt-Prkdc^em26Cd52^Il2rg^em26Cd22^/Gpt (NCG) mice (GemPharmatech Co., Ltd) were used for PDX studies. Fresh tumor tissue fragments of renal clear cell carcinoma were collected with the informed consent in October 2019 from a 58-year-old male who was diagnosed with renal clear cell carcinoma at the First Affiliated Hospital of Sun Yat-sen University and underwent radical nephrectomy. The primary tumor is renal clear cell carcinoma as diagnosed based on the surgical pathology report (Supplementary fig. 10). The tumor size is about 12 cm × 11 cm × 9 cm. The CT scan image of the kidney showed that the maximum diameter of the tumor is also about 12 cm (Supplementary fig. 11). The tumor tissue was cut into several pieces of ~3 mm × 2 mm × 2 mm (ca 10–15 mm^3^) and then transplanted subcutaneously in the flanks of male NCG mice (P0). The P0 tumors were grown and subsequently transplanted in mice to make P1, P2 and P3 PDX. P3 PDX were intratumorally injected with control lentiviruses (NC) or lentiviruses expressing LZTFL1 (10^9^ IU/ml) in a volume of 20 μL/100 mm^3^ tumor volume (2–3 sites per tumor) twice a week. The xenograft volume was measured weekly using the following formula: V = 0.5 × length × width^2^. Mice were sacrificed after 5 weeks. Tumors were excised, weighted, and subjected to histological and biochemical analysis.

No sample size calculations were conducted for animal experiments. We determined sample size according to our experience and previous literature. No data was excluded from the experiments. Randomization was applied to determine how animals were assigned to indicated groups. To achieve randomization, mice were numbered by body weight. Then random number table was used to assign animals to indicated experimental groups, and no blinding was done.

### Statistical analysis

Statistical analysis for SYSU cohorts was carried out with R software (version 4.1.1, http://www.R-38 project.org). The *χ*2 test was used to assess the statistical significance of the association of the expression of LZTFL1 with the patient’s clinicopathologic parameters and its correlation. Univariate and multivariate analysis was performed using the Cox proportional hazards model. Comparisons between groups for statistical significance were performed with the independent sample *t*-test. Variance was similar between groups that were statistically compared. Correlations were analyzed by Pearson correlation test. All other statistical analyses were carried out using GraphPad Prism v.6.0. Survival analysis was performed by Kaplan–Meier curves and log-rank test for significance. *P* values of <0.05 were considered statistically significant.

## Supplementary information

Supplementary material

## Data Availability

All relevant data are available from the authors upon request.
