# Supplementary material for: LZTFL1 inhibits kidney tumor cell growth by destabilizing AKT through ZNRF1-mediated ubiquitin proteosome pathway
Source: Oncogene. 2023 Mar 25;42(19):1543–57. doi: 10.1038/s41388-023-02666-x (PMC10039360; doi:10.1038/s41388-023-02666-x)
Supplement: Supplementary file 1 — Supplementary material [file 41388_2023_2666_MOESM1_ESM.pdf]

Supplemental methods.

CCLE mRNA expression and CNV data of 29 renal carcinoma cell lines were downloaded from CCLE (Cancer Cell Line Encyclopedia) database (http://ualcan.path.uab.edu/analysis-prot.html).

RNA isolation and quantitative real-time PCR (qRT-PCR)

Total RNA was extracted using Total RNA Extraction Reagent (EZBioscience-TZ1, China) following the instructions. A260/A280 ratio was measured by NanoDrop to determine RNA concentration. PrimeScript RT reagent kit (EZBioscience, China) was used to perform cDNA synthesis, and SYBR Green PCR reagent (EZBioscience, China) was used to further conduct qRT-PCR according to the protocol. Reverse transcription reaction conditions: reaction at 42 ° C for 15min, 95 ° C for 30s, the product obtained at the end of the reaction is cDNA. For qRT-PCR reaction system: 5 minutes of incubation at 95 °C followed by 40 cycles of 10 seconds at 95 °C and 30 seconds at 60 °C. GAPDH was used as an internal control. The PCR primer sequences were exhibited in supplementary table 2. The 2-ΔΔCT relative quantification method was used to analyze the data.

Transwell assays

Transwell migration assays were performed using a 24-well transwell chamber (Corning, NY, USA). About 50,000 and 100,000 cells were resuspended in serum-free medium and seeded onto the upper chamber separately, and the lower chamber was added with 10% FBS-containing medium as the chemo-attractant. 12 hrs later, migrated cells were fixed, sained, and counted under a light microscope.

PDX Immunofluorescence

Paraffin sections of PDX tumors were baked in an oven at 65 °C for 2 h. Paraffin sections were then deparaffinized in xylene and rehydrated in graded ethanol. EDTA antigen retrieval solution (Beijing Zhong Shan Goldenbridge Biotechnology Company Ltd, ZLI-9069) was used to perform antigen retrieval. After that, the sections were treated with 3% H_2O_2 to block the endogenous peroxidase activity, permeabilization was performed using 0.5%Triton X-100 (Beijing Zhong Shan Goldenbridge Biotechnology Company Ltd, ZLI-9308) for 20min at room temperature, followed by incubating in blocking buffer (5% bovine serum albumin). Anti-GFP antibody (Proteintech, #66002-1-Ig) was added on the sections and incubated in a humidified chamber at 4°C overnight. Following that, goat anti-rabbit/mouse IgG conjugated horseradish

peroxidase was added to the sections (Beijing Zhong Shan Goldenbridge Biotechnology Company Ltd, PV-6000) for 20 min at room temperature. The slides were sealed with fluorescence sealant (containing DAPI) (Beijing Zhong Shan Goldenbridge Biotechnology Company Ltd, ZLI-9557), then observed, counted and photographed under a fluorescence microscope.

Supplemental figures

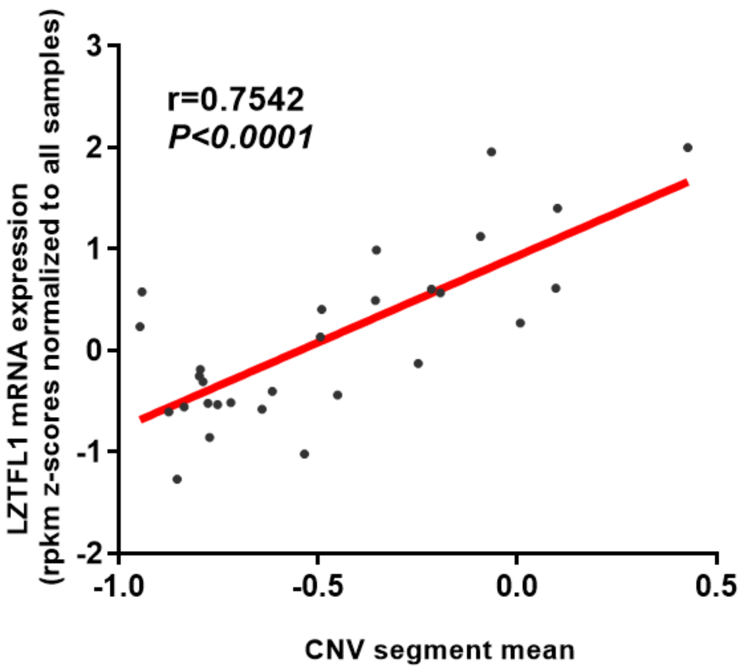

Supplemental figure 1. Correlation of LZTFL1 mRNA expression level and CNV segment mean data of 29 renal cell carcinoma cell lines in Cancer Cell Line Encyclopedia (CCLE) (https://sites.broadinstitute.org/ccle/datasets).

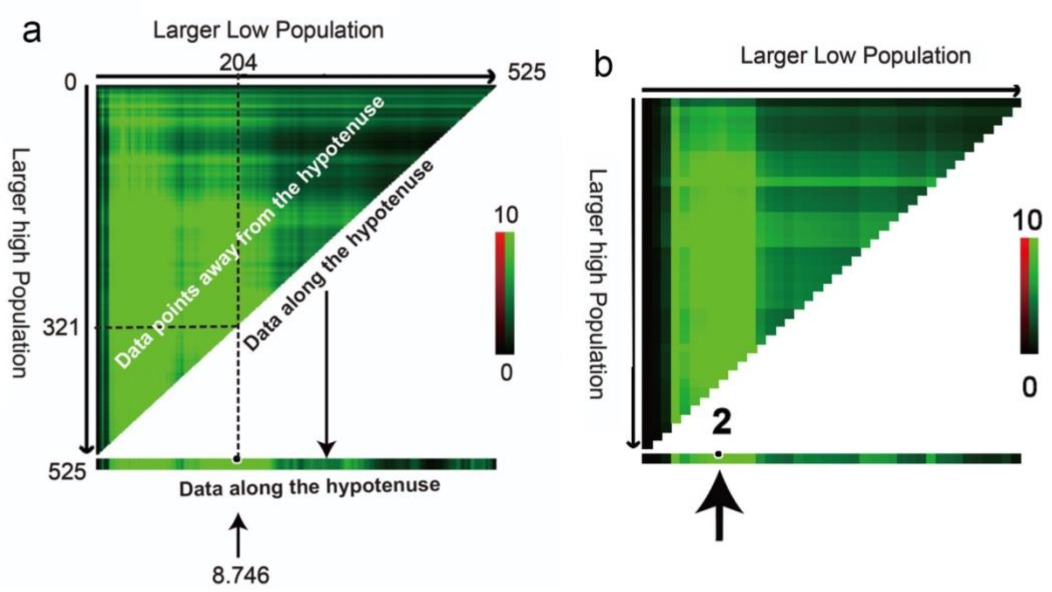

Supplemental figure 2. (a) X-tile plot of mRNA of LZTFL1 from the TCGA database. (b) X-tile plot of IHC scores of ccRCC tissue microarray. The vertical axis of the X-tile plots represents all possible "high" populations, with the size of the high population increasing from top to bottom. The horizontal axis represents all possible "low" populations, with the size of the low population increasing from left to right. The arrows represent the direction in which the low subset (X-axis) and the high subset (Y-axis) increase in size. The criterion for selecting the best cut-point is based on the most significant division (minimum *P*-value) (PMID: 15534099). The best cut-point is when LZTFL1 mRNA expression (mRNA RPMK, log2) is equal to 8.746 (a). Data was then divided into high(n=321) or low(n=204) subsets.

The bar below the hypotenuse is the same with data along the hypotenuse. Data along the hypotenuse represent results from a single cut-point that divides the data into high or low subsets. Data points away from the hypotenuse up or to the left represent results from two cut-points that define an additional "middle" population in addition to the high and low subsets. Each point (pixel) represents the data from a given set of divisions. A χ^2 value is calculated for every possible division of the population shown on the grid using a color code. Coloration of the plot represents the strength of the association at each division, ranging from low (dark, black) to high (bright, green, or red). Red coloration indicates an inverse correlation with survival, whereas green coloration represents direct associations. The optimal cut-point occurs at the brightest pixel (green or red).

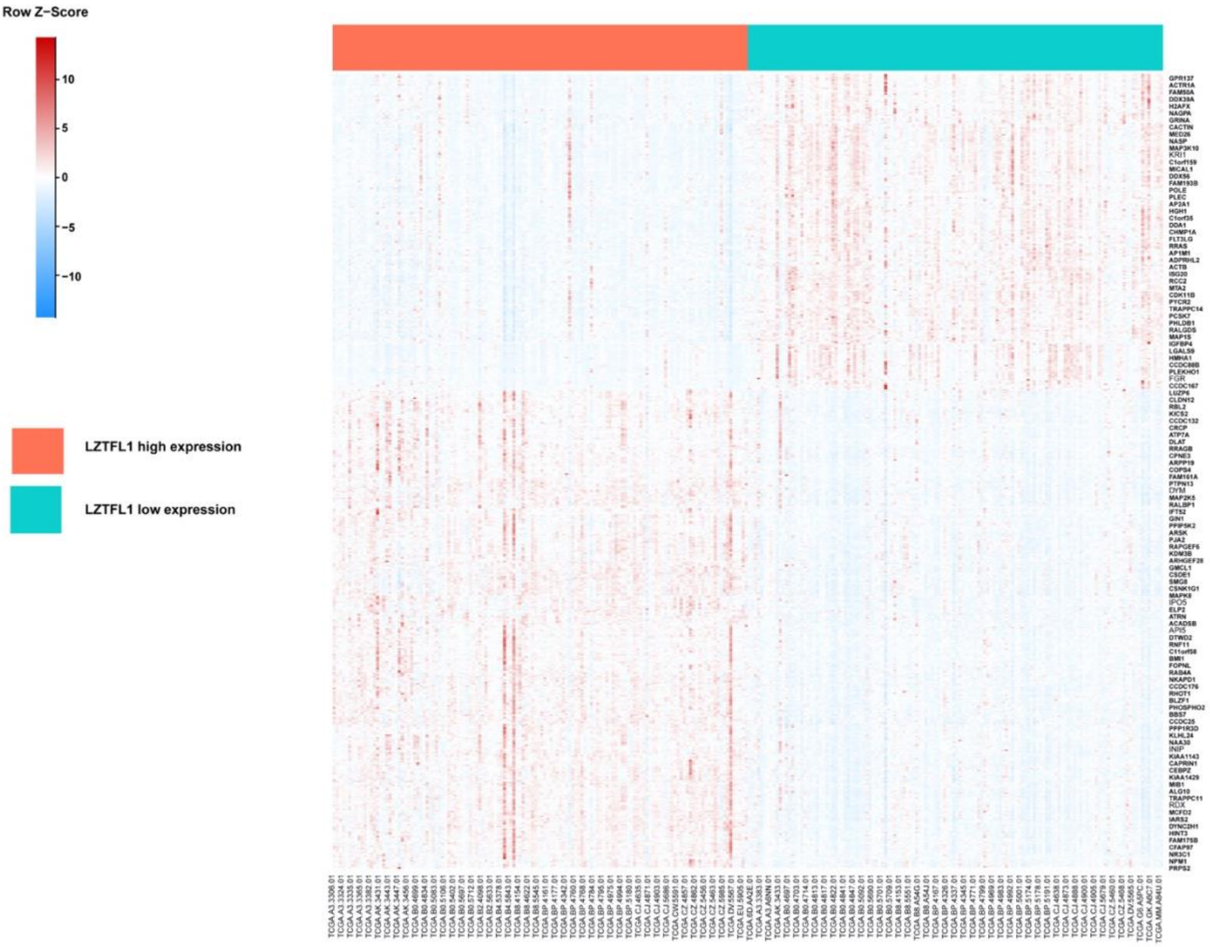

Supplemental figure 3. Heatmap of differentially expressed genes in low and high LZTFL1-expressing groups in TCGA database. A heatmap showing unsupervised hierarchical clustering of gene expression identified by analyzing LZTFL1 low and LZTFL1 high tumors in the TCGA-ccRCC cohort.

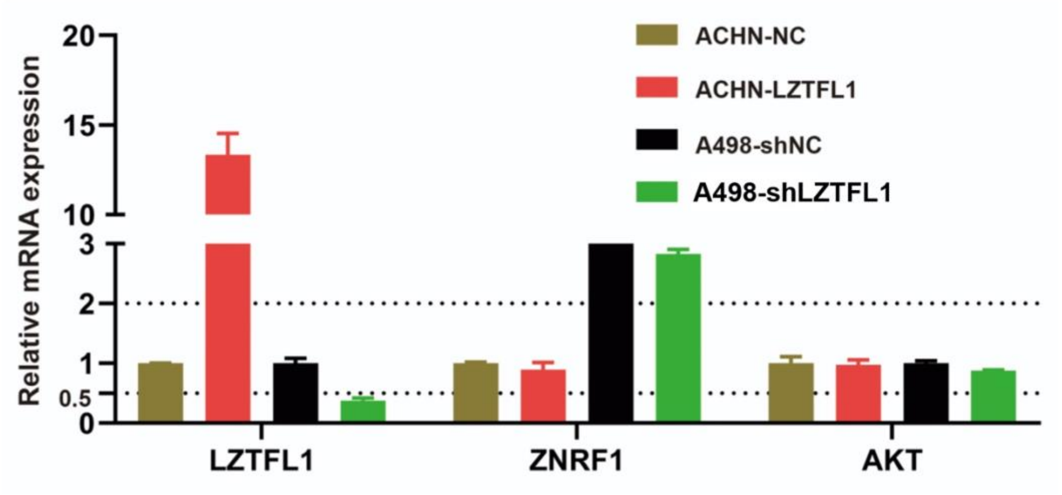

Supplemental figure 4. Relative mRNA of LZTFL1, ZNRF1, and AKT in cells that overexpress LZTFL1 (ACHN-LZTFL1) or LZTFL1 knockdown (A498-shLZTFL1), and their respective control cells.

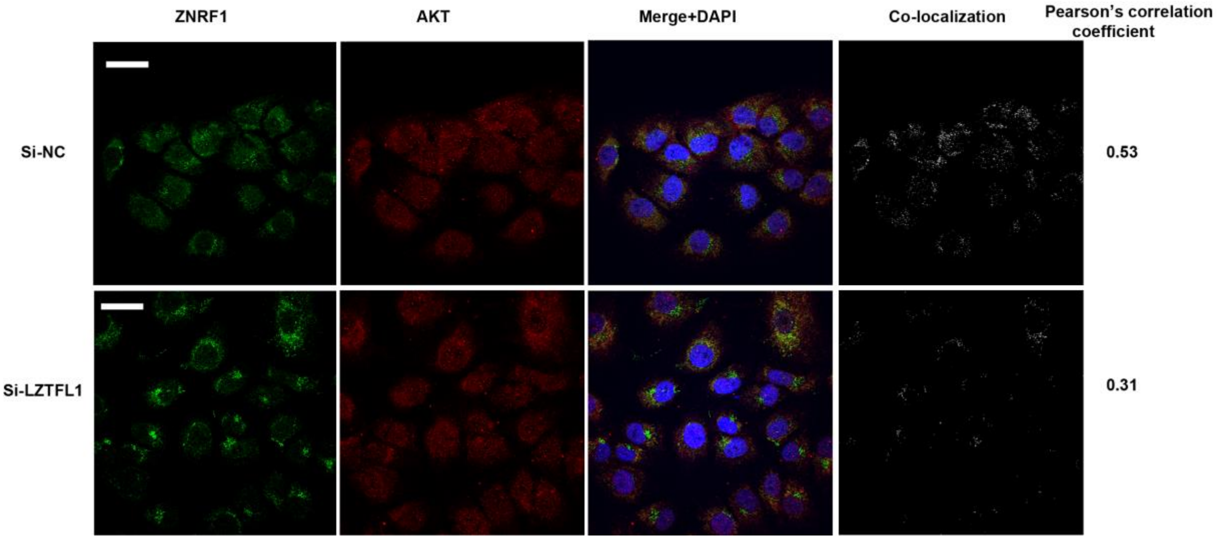

Supplemental figure 5. Immunofluorescence staining of ZNRF1 (green), AKT (red), and DAPI (blue) in A498 cells. Scale bar, 40 μm. A498 cells were transfected with control or LZTFL1 specific siRNA (si-LZTFL1). Then cells were fixed and stained with anti-ZNRF1 (ABP60990, Abbine,1:50) and anti-AKT antibody (#9272, CST, 1:100). Representative images were acquired using a fluorescence microscopy (OLYMPUS FV1000 confocal microscopy, Japan). A quantitative analysis of co-localization in confocal sections was obtained using the information obtained from scatterplots. Pearson's correlation coefficient is used to analyze the entire scatterplot, which is one of the standard techniques to describe the degree of overlap between the two images. The value is analyzed by OLYMPUS FV1000 software automatically. -1 represents a total lack of overlap between pixels from the images, and 1 indicates perfect image registration.

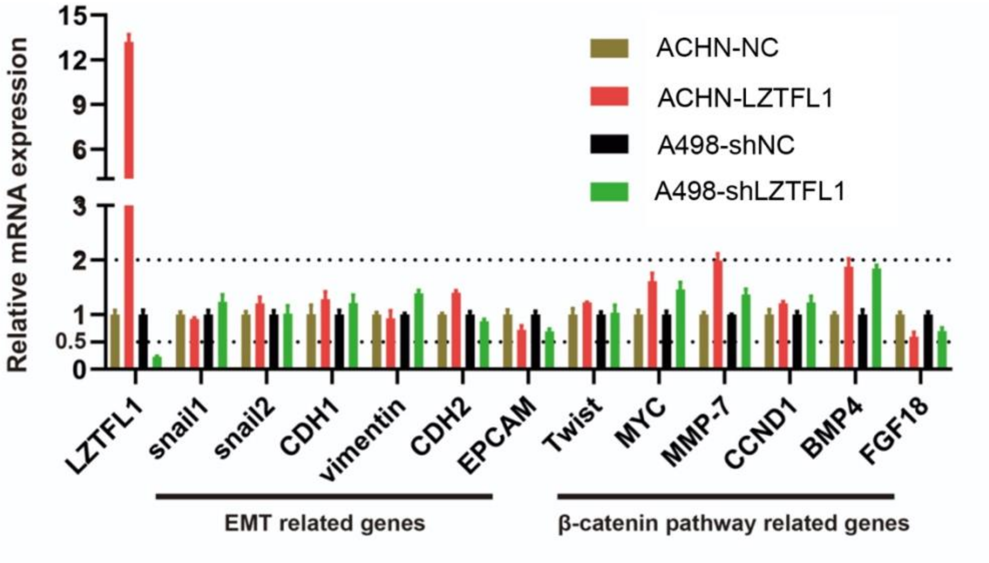

Supplemental figure 6. Relative mRNA of *LZTFL1* and genes related to EMT and β-catenin signaling pathway from cells overexpress LZTFL1 (ACHN-LZTFL1) or knockdown LZTFL1 (A498-shLZTFL1) and respective control cells.

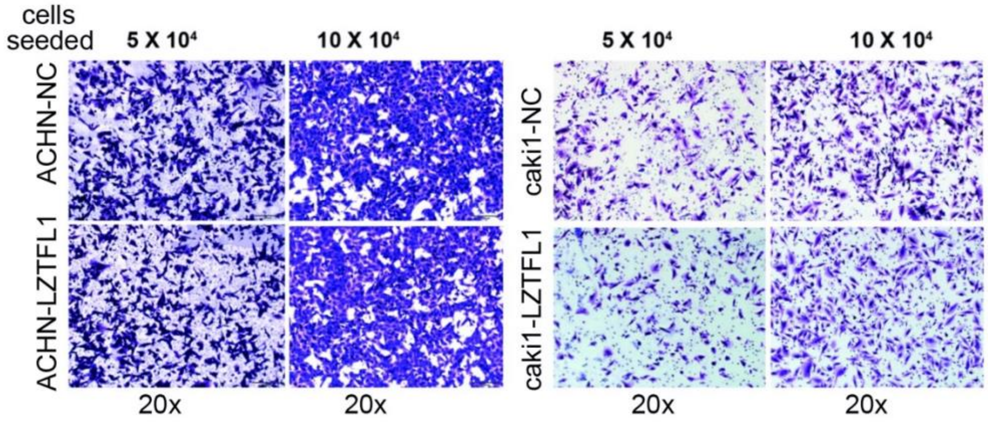

Supplemental figure 7. Crystal violet staining of cells as indicated on the underside of the microporous membrane after 12 h of incubation. ACHN and caki1 cells overexpress LZTFL1 or control vector (NC).

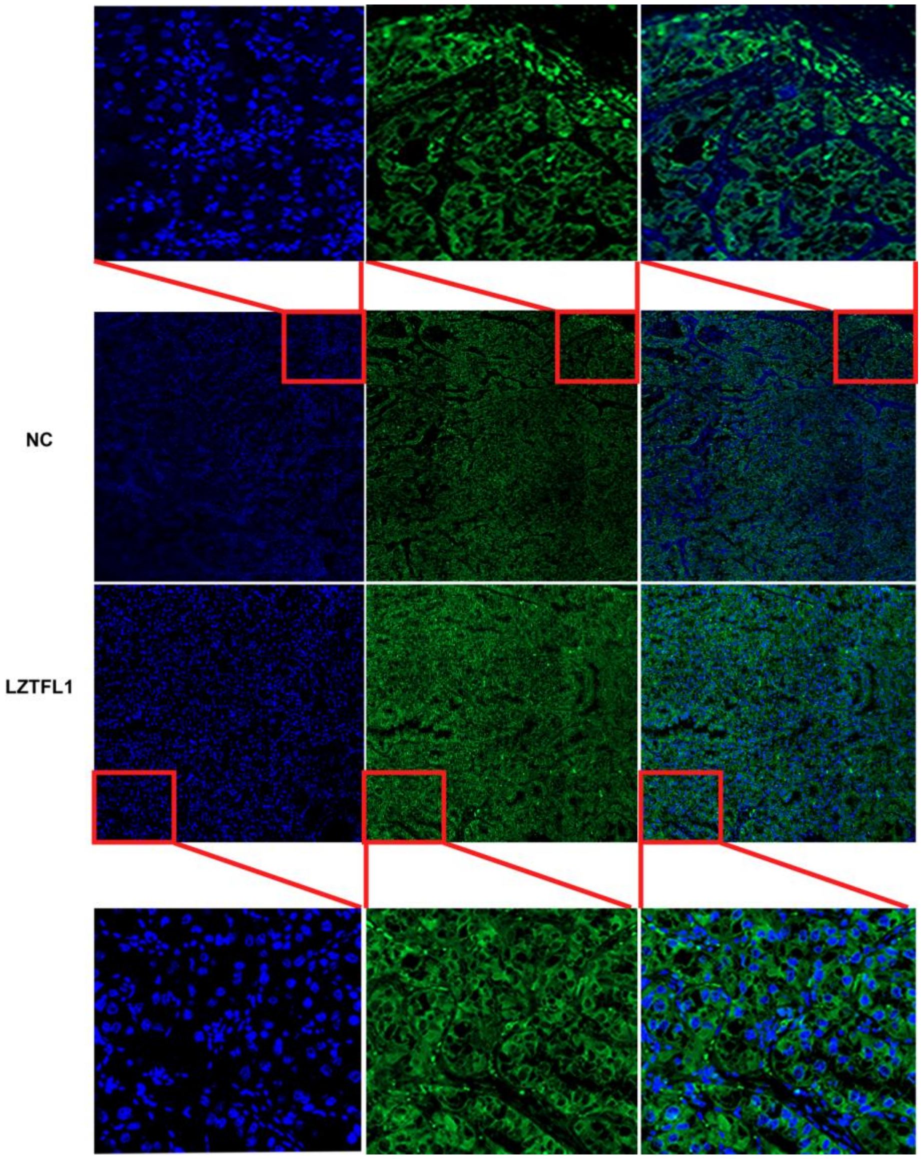

Supplemental figure 8. Immunofluorescence micrographs of GFP (green) and DAPI (blue) staining from PDXs transduced with control lentiviruses (NC) and lentiviruses expressing LZTFL1 (LZTFL1). GFP is expressed from a reporter gene on the lentiviral vector.

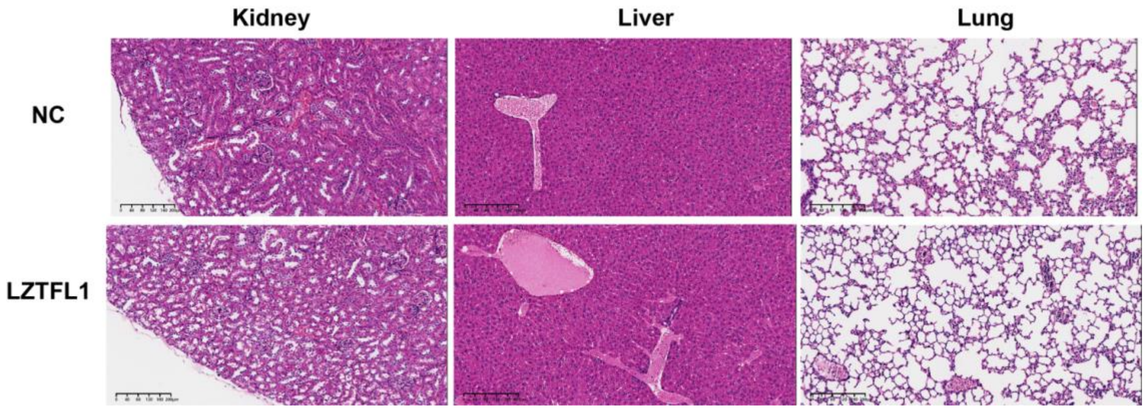

Supplemental figure 9. H&E staining of tissues from mice that bear PDXs treated with control lentiviruses (NC) or lentiviruses expressing LZTFL1.

病理活体组织诊断报告书

(Surgical Pathology Report)

病理号：P[redacted]
(Pathology number)

姓　　名：[redacted]　性别：男 (Male)　年龄：58岁 (Age:58)　住 院 号：[redacted]

送检医院：中山大学附一院　送检科室：泌尿外科二区　床　　号：029

标本类型：大标本　送检医生：[redacted]　收到日期：2019-10-25　17:04

临床诊断：右肾癌并腔静脉癌栓 (Preoperative diagnosis：Renal clear cell carcinoma with vena caval tumor thrombus)

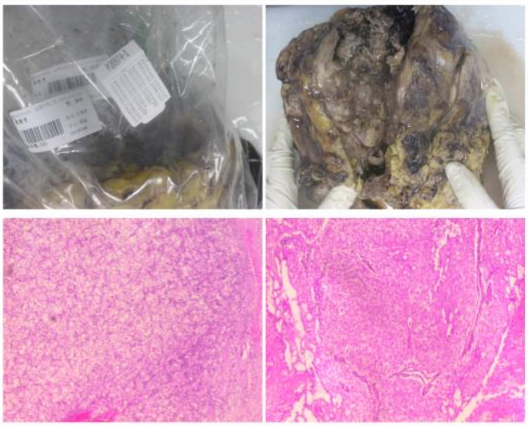

肉眼所见：

1.（右肾）送检肾组织一块，大小17×16×9cm，切开可见一个12×11×9cm灰黄灰白肿物，局部坏死，输尿管长3cm，直径0.3cm。
2.（癌栓）送检暗红组织一堆，直径3cm。
3.（腹膜后淋巴结）送检灰褐组织一堆，直径2cm，全埋制片。

病理诊断：

1.（右）肾透明细胞癌，核级3级，伴坏死及炎症细胞浸润，肾周脂肪未见癌，输尿管切缘未见癌。
免疫组化：癌细胞CK（+），Vimentin部分（+），CD10（+），CAIX（+），PAX-8部分（+），E-cadherin弱（+），CD117（-），CK7（-）。
2. 为癌组织及血块。
3.（腹膜后淋巴结）淋巴结未见癌（0/3）。

Specimen received：

1.(Right renal)The specimen is kidney tissue，and the Size is 17 X 16 X 9cm。After incision，a grayish yellow tumor is inside，partial necrosis ,and the size is 12X11X9cm.The length of ureter is 3cm, and diameter is 0.3cm.
2.(tumour thrombus)The specimen is dark red tissue,and diameter is 3cm.
3.(retroperitoneal lymph node)The specimen is taupe tissue,and diameter is 2cm.

Final microscopic Diagnosis：

1.(Right renal)renal clear cell carcinoma,Fuhrman Nuclear Grade 3, partial necrosis and inflammatory cell infiltration.
IHC:CK(+)，Vimentin partial (+)，CD10(+),CAIX(+),PAX-8 partial (+),E-cadherin weak(+) ,CD117(-),CK7(-)
2.renal clear cell carcinoma tissue and blood clot.
3.(retroperitoneal lymph node)No tumor tissue is found in lymph nodes(0/3).

报告医生：[redacted]　签名：　报告日期：2019-11-05

地址：广州市中山二路58号　邮编：510080　电话：87755766-8546　共1页/第1页

Supplemental figure 10. Surgical pathology report of tumor tissue from a patient diagnosed with kidney cancer.

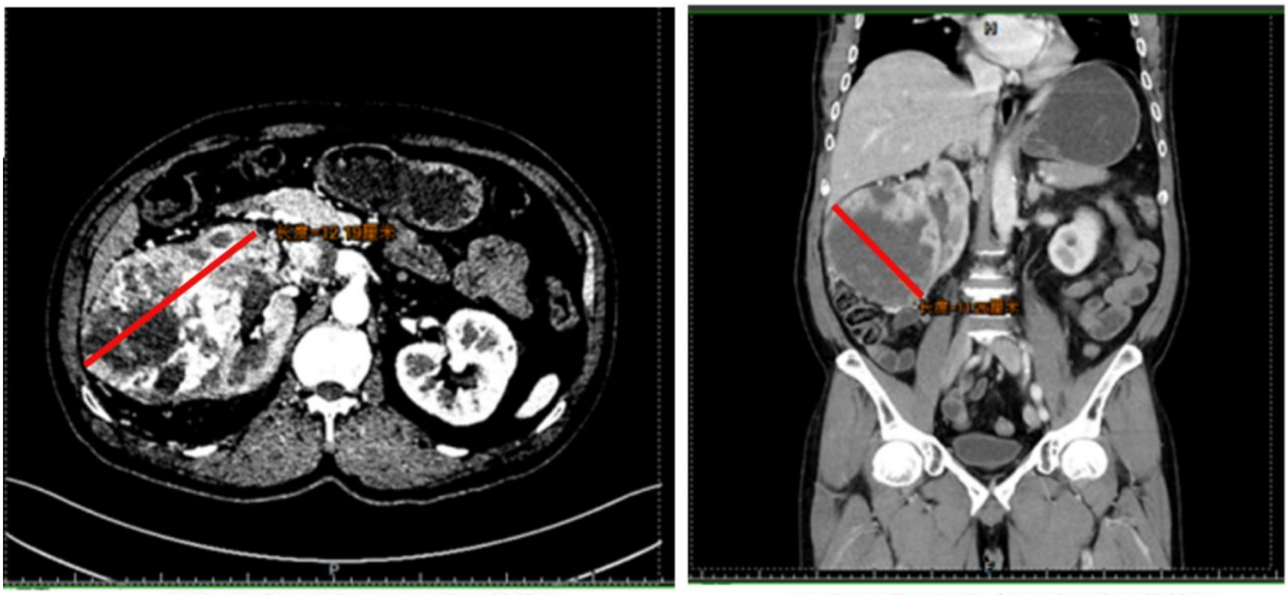

maximum diameter in cross section=12.19cm

maximum diameter in Coronal section=11.26cm

Supplemental figure 11. The CT scan image of the kidney of a patient, showing that the maximum diameter of the tumor is about 12cm.

Supplementary table 1: List of TCGA cancer types.

Abbreviations	Cancer Type	Tumor	Normal
**BLCA**	Bladder Urothelial Carcinoma	408	19
**BRCA**	Breast invasive carcinoma	1097	114
**CESC**	Cervical Squamous Cell Carcinoma and Endocervical Adenocarcinoma	305	3
**CHOL**	Cholangiocarcinoma	36	9
**COAD**	Colon adenocarcinoma	286	41
**ESCA**	Esophageal Carcinoma	184	11
**GBM**	Glioblastoma Multiforme	156	5
**HNSC**	Head and Neck Squamous Cell Carcinoma	520	44
**KICH**	Kidney Chromophobe	67	25
**KIRC**	Kidney Renal Clear Cell Carcinoma	533	72
**KIRP**	Kidney Renal Papillary Cell Carcinoma	290	32
**LIHC**	Hepatocellular Carcinoma	371	50
**LUAD**	Lung Adenocarcinoma	515	59
**LUSC**	Lung Squamous Cell Carcinoma	503	52
**PAAD**	Pancreatic Adenocarcinoma	178	4
**PCPG**	Pheochromocytoma and Paraganglioma	179	3
**PRAD**	Prostate Adenocarcinoma	497	52
**READ**	Rectum adenocarcinoma	166	10
**SARC**	Sarcoma	260	2
**SKCM**	Skin Cutaneous Melanoma	472	1
**STAD**	Stomach Adenocarcinoma	415	34
**THCA**	Thyroid Carcinoma	505	59
**THYM**	Thymoma	120	2
**UCEC**	Uterine Corpus Endometrial Carcinoma	546	35

Supplemental table 2: siRNA and shRNA sequences, and RT-PCR primers.

siRNA (target sequence)	
siZNRF1	GCCGTACTGTCAACCTCAA
siLZTFL1	GCCTAAATGAGCACCATCAAA
shRNA (target sequence)	
shLZTFL1-1	GCCTAAATGAGCACCATCAAA
shLZTFL1-2	GGTTCATAGTGAGGTGGAATC
RT-PCR primers	
CDH1	Forward: 5’-CGAGAGCTACACGTTCACGG-3’<br>Reverse: 5’-GGGTGTCGAGGGAAAAATAGG-3’
CDH2	Forward: 5’-TCAGGCGTCTGTAGAGGCTT-3’<br>Reverse: 5’-ATGCACATCCTTCGATAAGACTG-3’
Vimentin	Forward: 5’-GACGCCATCAACACCGAGTT-3’<br>Reverse: 5’-CTTTGTCGTTGGTTAGCTGGT-3’
EPCAM	Forward: 5’-AATCGTCAATGCCAGTGTACTT-3’<br>Reverse: 5’-TCTCATCGCAGTCAGGATCATAA-3’
Twist	Forward: 5’-GTCCGCAGTCTTACGAGGAG-3’<br>Reverse: 5’-GCTTGAGGGTCTGAATCTTGCT-3’
MYC	Forward: 5’-GGCTCCTGGCAAAAGGTCA-3’<br>Reverse: 5’-CTGCGTAGTTGTGCTGATGT-3’
MMP7	Forward: 5’-GAGTGAGCTACAGTGGGAACA-3’<br>Reverse: 5’-CTATGACGCGGGAGTTTAACAT-3’
CCND1	Forward: 5’-GCTGCGAAGTGGAAACCATC-3’<br>Reverse: 5’-CCTCCTTCTGCACACATTTGAA-3’
BMP4	Forward: 5’-ATGATTCCTGGTAACCGAATGC-3’<br>Reverse: 5’-CCCCGTCTCAGGTATCAAACT-3’
FGF18	Forward: 5’-ACTTGCCTGTGTTTACACTTCC-3’<br>Reverse: 5’-GACCTGGATGTGTTTCCCACT-3’
AKT	Forward: 5’-AGCGACGTGGCTATTGTGAAG-3’<br>Reverse: 5’-GCCATCATTCTTGAGGAGGAAGT-3’
ZNRF1	Forward: 5’-TGGCAATGGTTACCAGGAGAC-3’<br>Reverse: 5’-GCAGAGGTAGAGCATCCGC-3’
